# Putting co-creation into practice: lessons learned from developing a midwife-led quality improvement intervention

**DOI:** 10.1080/16549716.2023.2275866

**Published:** 2023-11-06

**Authors:** Johanna Blomgren, Michael B. Wells, Kerstin Erlandsson, Dinah Amongin, Lydia Kabiri, Helena Lindgren

**Affiliations:** a Department of Women’s and Children’s Health, Karolinska Institutet, Stockholm, Sweden; b Institution of Health and Welfare, Dalarna University, Falun, Sweden; c Department of Health Policy Planning and Management, School of Public Health, College of Health Sciences, Makerere University, Kampala, Uganda; d Department of Nursing and Midwifery, School of Health Sciences, College of Health Sciences Makerere University, Kampala, Uganda; e Department of Health Promotion, Sophiahemmet University, Stockholm, Sweden

**Keywords:** Co-creation, midwifery, quality improvement, maternal health, evidence-to-practise

## Abstract

**Background:**

Integrating evidence-based midwifery practices improves healthcare quality for women and newborns, but an evidence-to-practice gap exists. Co-created quality improvement initiatives led by midwives could bridge this gap, prevent resource waste and ensure intervention relevance. However, how to co-create a midwife-led quality improvement intervention has not been scientifically explored.

**Objective:**

The objective of this study is to describe the co-creation process and explore the needs and determinants of a midwife-led quality improvement targeting evidence-based midwifery practices.

**Methods:**

A qualitative deductive approach using the Consolidated Framework for Advancing Implementation Science was employed. An analysis matrix based on the framework was developed, and the data were coded according to categories. Data were gathered from interviews, focus group discussions, observations and workshops. New mothers and birth companions (*n* = 19) were included through convenience sampling. Midwives (*n* = 26), professional association representatives, educators, policymakers, managers, and doctors (*n* = 7) were purposely sampled.

**Results:**

The co-creation process of the midwife-led Quality Improvement intervention took place in four stages. Firstly, core elements of the intervention were established, featuring a group of midwife champions leading a quality improvement initiative using a train-the-trainers approach. Secondly, the intervention needs, context and determinants were explored, which showed knowledge and skills gaps, a lack of shared goals among staff, and limited resources. However, there was clear relevance, compatibility, and mission alignment for a midwife-led quality improvement at all levels. Thirdly, during co-creation workshops with new mothers and companions, the consensus was to prioritise improved intrapartum support, while workshops with midwives identified enhancing the use of birth positions and perineal protection as key focus areas for the forthcoming Quality Improvement intervention. Lastly, the findings guided intervention strategies, including peer-assisted learning, using existing structures, developing educational material, and building stakeholder relationships.

**Conclusions:**

This study provides a practical example of a co-creation process for a midwife-led quality improvement intervention, which can be relevant in different maternity care settings.

## Introduction

Integrating evidence-based midwifery practises into healthcare systems can significantly improve the quality of care, well-being, and health outcomes for both women and newborns [1,2]. Evidence-based midwifery practises include elements such as providing continuous labour support and promoting upright birthing positions, relaxation techniques and skin-to-skin contact between mothers and babies [3]. Many of these practices are cost-effective as they reduce interventions and do not require expensive equipment, making them particularly relevant in settings with limited resources [4,5]. Integrating the latest evidence into maternal healthcare practices is often complex and poses frequent challenges [4]. Despite the critical role of evidence-based midwifery practises, deficiencies in enabling environments and supportive structures for midwives persist in many healthcare settings, resulting in an evidence-to-practice gap, ultimately resulting in sub-optimal midwifery care quality [1].

One way to close the evidence-to-practice gap is to empower and train healthcare professionals, like midwives, to lead quality improvement (QI) interventions [5,6]. A co-creation approach, which involves collaboration among academic researchers and stakeholders, is valuable for developing public health interventions, such as QIs [7]. This approach facilitates the identification of priorities, power-sharing, and knowledge generation from different sectors [7,8]. However, in numerous co-creation processes and healthcare improvements, key decision-makers, clinicians and end-users involvement are often overlooked, or their views are dismissed as resistant to change [9,10]. This oversight can lead to suboptimal intervention designs and the inefficient allocation of resources, time, and effort [9–11]. For instance, Chalmers and Glasziou [12] estimate that 85% of funding for health research is wasted on poor intervention designs that address questions and outcomes of minor relevance to clinicians and end-users. Therefore, employing a framework to guide the selection of relevant stakeholders is often beneficial [13,14].

The Midwize framework is developed to help guide the selection of stakeholders to include in change processes’ in maternal healthcare [13]. The framework advocates for midwives as change agents with a strong emphasis on fostering collaboration among the education, regulation (including policy level), professional associations, civil society (including healthcare end-users and community members) and clinical sectors [13].

To our knowledge, there is a lack of scientific exploration regarding the process and outcomes when midwives, end-users, and other relevant stakeholders co-create a QI initiative to close the evidence-to-practice gap in midwifery. Therefore, this study aims to investigate the process of co-creating a midwife-led quality improvement intervention targeting the evidence-to-practice gap within midwifery and learn how to effectively apply this across different settings. Its specific objectives are to describe the co-creation process and explore the needs and determinants of a midwife-led QI targeting evidence-based midwifery practices.

## Method

### Study setting

The study took place at a public national referral hospital in Uganda. The maternity unit has 32 midwives, handles around 9,000 births annually, and has a caesarean-section rate of 50%. Hospital management has declared a willingness to engage in QI projects to improve midwifery practices and maternal and newborn healthcare.

In Uganda, a QI approach links the Ministry of Health with healthcare facility managers [15]. This initiative has increased nationwide maternal and newborn healthcare standards [16,17]. However, midwife-led QI interventions are rare in Uganda and crucial aspects of evidence-based midwifery practices, such as informed decision-making, respectful care and proper monitoring, still need improvements [18,19].

### Study design and conceptual framework

A qualitative approach using the Consolidated Framework for Advancing Implementation Science (CFIR) [20] was used to guide the co-creation of the intervention (i.e. the co-creation process). The CFIR is a theory-based guide which helps identify and explore determinants (i.e. barriers and facilitators) to implementation and tailor intervention strategies. The framework consists of five domains (Intervention Characteristics, Outer Setting, Inner Setting, Individuals, and Process) and multiple constructs within each domain, providing a systematic way to evaluate and optimise implementation efforts.

### Participants and sampling

Participants for semi-structured interviews were sampled purposely to represent multiple vital sectors for creating change within clinical midwifery care according to the Midwize framework [13]: one leader within the regulation (policy) sector at the Uganda Ministry of Health, one midwife from The National Midwives Association of Uganda, two academic professionals working in midwife education programmes and three stakeholders at the clinical level, two obstetricians and one midwife manager. The informants were approached via email, and all six who were targeted agreed to participate. A convenience sampling was made to capture a momentary cross-section of the hospital’s labour ward clientele, comprising new mothers and their birth companions discharged on the day of a focus group discussion (FGD). This sampling strategy yielded a diverse sample; all new mothers (*n* = 15) and birth companions, two sisters, one friend and one grandmother (*n* = 4) agreed to participate. To ensure an inclusive intervention design, we purposely invited all 32 midwives employed at the hospital to participate in one of the three FGDs. Five midwives did not attend due to time constraints at the workplace, leaving 26 participants.

### Data collection

Gathering data unfolded across four stages, aligning with the co-creation approach.

#### First stage – defining core elements

To identify the upcoming QI intervention core elements, i.e. the essential and indispensable elements of an intervention [20], the research group studied lessons learned from providing an online capacity-building programme called Midwize, which focuses on QI in midwifery [21]. In addition, a non-systematic literature review on co-creation methods, midwifery quality of care, and intervention design was used to guide the selection of core components of the intervention. This stage helped ensure an evidence-based approach and clarified which elements are crucial for success and which can be modified without compromising outcomes [22].

#### Second stage – exploring needs, context and determinants

In the second stage of the intervention design, we used interviews, FGDs and environmental observations guided by the CFIR framework to explore needs, context and determinants of the upcoming midwife-led QI. First, environmental observations were conducted at the hospital’s maternity unit to understand the current health problems, identify symptoms and causes of inadequate quality, and identify factors that could impact the intervention [23,24]. The three researchers, all certified midwives with over 15 years of clinical experience and a history of conducting environmental observations in similar settings carried out intermittent 30–45-min-long observations over three days during the daytime. The researchers sat, stood, or moved about while observing ongoings in labour, admissions, antenatal care, and hospital corridors. Notes were made on an observation sheet to document what they saw, heard and felt. The form was kept simple. On one side of the form, they recorded what they saw and heard, while on the other side, they noted their reflections and thoughts [23]. Following the observations, the authors convened to discuss their overall observations and strategise feedback to the staff.

Semi-structured interviews based on the CFIR's Interview Guide tool [25] were then held with multisectoral stakeholders to explore determinants affecting the upcoming midwife-led QI. We mainly focused on partnerships, connections, policies, laws, available resources, compatibility and work infrastructure. Four interviews were held online via Zoom and two at the informants’ offices.

Three FGDs with midwives based on the CFIR's Interview Guide tool [25] were held to explore midwifery practices current status and needs, available resources, compatibility, capabilities, and work infrastructure.

Finally, two FGDs were held to explore the end-users, i.e. women who recently gave birth and their birth companions’ experiences with the care from hospital admission to discharge and their needs.

#### Third stage - co-creation workshops

Following the FGDs conducted with end-users and midwives, co-creation workshops were organised with the same participants and researchers. The workshops were designed to expand upon the insights gathered from the FGDs, interviews, and observations, further exploring the identified needs and contextual factors discovered in the earlier stages and creating a user-friendly, relevant, acceptable, and sustainable intervention [14,23]. The ultimate goal of these workshops was to make informed decisions about the specific evidence-based midwifery components that should be the focus of the QI intervention.

At the two workshops with end-users, we first identified suggested ‘touchpoints,’ i.e. critical moments concerning the service and improving the quality of midwifery care. Secondly, these touchpoints were prioritised by the group. Lastly, suggestions for areas to focus on in the upcoming QI were decided among the end-users.

At the three workshops with midwives, sessions started with framing the topic and defining evidence-based midwifery practises based on The Lancet Commission Series on Midwifery [3]. Secondly, the intervention’s core and adaptable elements (as defined in stage one) and current effective and ineffective practices derived from the conducted environmental observations, interviews and FGD (second stage) were shared. Finally, touchpoints for the upcoming QI were identified, prioritised and decided on in each workshop.

All FGDs and workshops were held in a separate room at the hospital where only the researcher and midwives or the end-users were present. Interviews, FGDs and workshops were conducted by four authors with experience in co-creation and QI. All sessions with midwives and all interviews were in English, as all informants spoke English fluently. The sessions with end-users were held concurrently in English and one of the local languages, Luganda. The sessions with the end-users were held for 62–72 min (mean = 67 min), the midwives’ sessions ranged from 75–130 min (mean = 108 min), and the interviews ranged from 26–55 min (mean = 38 min). All interviews, FGDs and workshops were audio recorded and transcribed. After analysing the total data entry from environmental observations, interviews, FGDs and workshops, saturation was considered achieved.

#### Fourth stage – finalising the intervention design

Four of the authors and a group of seven midwives working at the hospital who wished to lead the QI work, the so-called ‘Midwize Ambassadors’, made the final analysis of the determinants of the midwife-led QI intervention, and outlined roles and responsibilities, initial strategies, dose and temporality based on the findings. The choice of strategies was influenced by the Expert Recommendations for Implementing Change (ERIC) [26], a compilation of strategies commonly used in combination with CFIR to help users identify effective strategies based on the outcomes of a context assessment [27]. The ERIC strategies have been developed by a panel of experts in implementation science and clinical practice to establish a consensus on standardised terminology, definitions, and categories for implementation strategies [28]. This fourth stage of the co-creation process was undertaken to formulate a clear and detailed plan with task assignments to facilitate a successful implementation [20].

### Data analysis

A deductive content analysis approach, guided by Elo and Kyngäs [29], using CFIR domains and constructs [20] was employed to systematically explore the midwife-led QI intervention’s contextual factors, needs, and determinants. Firstly, an analysis matrix based on CFIR was developed. Secondly, in the preparation phase, we read and re-read the transcribed text from FGDs, interviews, and notes from the environmental observations concurrently to get a complete picture of the data. Notes were written in the text to describe all aspects of the content while reading it. Thirdly, the data were coded according to the domain and categories of CFIR. Some domains and constructs were omitted to simplify the analysis. Table 1 illustrates the use of the CFIR domains and constructs related to the co-creation process of the midwife-led QI intervention.

**Table 1.** Categorisation matrix based on the Consolidated framework for Advancing implementation Science showing the application of domains and constructs [17].

CFIR domains	CFIR constructs	CFIR descriptions and application in this study
**Implementation Process**	Assessing needs and context	Priorities, preferences, and needs of the end-users and clinicians to guide the co-creation. Assessments should consider all contextual factors, both modifiable and non-modifiable
Activities on a spectrum from bottom-up grassroots or top-down mandated change efforts.		
**Inner Setting and Individuals**	Available Resources	Resources such as physical space, equipment, supplies and human resources needed to implement and deliver the intervention.
The setting in which the innovation is implemented and the roles and characteristics of individuals.	Compatibility	Compatibility with the setting and context, for example, does it fit workflows, goals and current processes?
	Capabilities	Individual(s) interpersonal capabilities, competence, knowledge, willingness and skills to fulfil roles and adopt the intervention.
	Work infrastructure	The organisation of tasks and responsibilities within and between individuals and teams
		General staffing levels.
**Outer setting**	Partnerships & Connections	If and how the Inner Setting is networked with external entities, including academic affiliations and professional organization networks.
The setting in which the Inner Setting exists. Captures macro-level factors that emanate from outside to the Inner Setting	Policies & Laws	If policies, legislation, regulations, professional group guidelines and recommendations support the intervention’s implementation and/or delivery.

## Results

### First stage - defining core elements

After the non-systematic review of the literature and exploring lessons learnt from the Midwize online capacity-building programme [21], four core elements of the QI intervention were determined:

1. The intervention will be led by a group of midwife champions (referred to as “Midwize ambassadors) based on its validated effectiveness in supporting interventions’ implementation, sustainability, and scale-up [26,30].
2. To ensure credibility, the intervention should focus on evidence-based midwifery practices, drawing upon The Lancet Commission’s series on midwifery [25]
3. Stakeholders from sectors suggested by the Midwize framework (education, regulation, professional associations, civil society and clinical sites) should be involved in creating and implementing the QI intervention to maximise user-friendliness, relevance, acceptability, feasibility and sustainability [13].
4. Ongoing training using a ‘train the trainers’ approach to create a collaborative learning environment which enhances the utilisation of the intervention [26,31] should be given on:
  - QI skills
  - Practical skills (based on the outcomes of the co-creation process)

Since the core elements were essential and indispensable, these four aspects would not be further discussed during the co-creation process, but instead used to help guide the co-creation conversations.

### Second stage - exploring needs, context and determinants

#### Needs and context

In the FGDs with new mothers and birth companions, several participants expressed satisfaction with the care they received during childbirth and praised the midwives for their competent handling and supportive presence. Similarly, during FGDs with midwives, some emphasised the significance of providing care with warmth and compassion to make women feel heard, understood, and at ease. However, according to several new mothers and birth companions, there was a lack of emotional and physical support during labour and birth at the hospital. One new mother shared that she was told to ’*Keep quiet. Labour pain is not new to us. We have seen that before.’* (New Mother #1)

Overall, new mothers and birth companions did not expect a high level of emotional and physical support from the staff before coming to the hospital, and women said they were happy as long as they and their babies were healthy. Many midwives and doctors were also aware of women’s low expectations of supportive care and also described a lack of evidence-based and respectful care at the hospital. For example, Midwife #4 said, *‘Most of them really appreciated the care … However, some of them also complain about some form of disrespect, especially when there is too much workload and people are tired. Sometimes, it may sound rude*.’

During the FGD with new mothers and birth companions, concerns were raised about the reputation of intrapartum care provided by the hospitals in the area. Issues such as cleanliness, limited space, lack of supportive care, intrusion of bodily autonomy, and high intervention rates led many to discourage giving birth at hospitals. A policy-level representative echoed these concerns and additionally emphasised cultural and normative factors to consider in the midwife-led QI intervention. Examples included physical abuse, such as slapping a mother, which in some settings can be seen as acceptable to enforce compliance with existing birthing norms of being quiet and giving birth in the supine position. The need to address such norms and cultures in the upcoming QI intervention, shift focus towards respectful and informative communication, and enhance women’s decision-making was highlighted. All women in the FGD described giving birth in the supine position without receiving information or recommendations for alternative positions.

Overall, new mothers and birth companions highlighted the importance of receiving respectful communication, information and advice from staff during labour and birth. One participant said: *‘I am not saying the midwives are bad, but they should think of other strategies to communicate … if you shout at someone who is in pain, it is not good’* (New mother #4). Some new mothers desired more pain relief and comforting measures during birth and labour. Some did not believe the midwives could alleviate pain under any circumstance, while most new mothers could imagine that massage or strokes could have lowered the pain. One new mother shared how the pain was easier to manage during her birth when a midwife and birth companion attended and supported her. In addition, some new mothers highlighted the value of support from students, as they had more time to give reassurance and communicate and explain procedures. Almost all new mothers wanted to have a birth companion present during birth. Many midwives also considered this significant and believed that mothers should be allowed to come with a person of their choice, even if this was not the standard practice at the hospital.

During the environmental observations, while many midwifery practices were meeting standards, some practices were not performed according to evidence and national or international guidelines, including women’s decision-making, the involvement of birth companions, perineal protection, usage of favourable birth positions, skin-to-skin care, information and communication and physical and emotional support.

#### Determinants

Midwives commonly described their job with words like ‘love’ and ‘passion’. However, many expressed barriers to QI due to an overwhelming patient load, organisational setup, long working hours and the frequent arrival of new colleagues and students. *‘The constraints in space make it difficult to implement most of the things you want to do,’* said midwife #8. On the other hand, midwives expressed that they are used to working with limited resources. One midwife (#10) said, *‘We always try our best in this space and what we have.’* After discussions in the group, many midwives concurred that most evidence-based midwifery practices can be done with minimal space and funds or by adopting new working methods.

Midwives and doctors described colleagues who might be hesitant to change and that it was crucial to start with the ones who were positive to encourage a broader enthusiasm. The policy-level representative raised the same issues and feared that clinical staff would resist change due to various factors, including insufficient knowledge, resources, time, and organisational structures. Among the academic representatives, the resistance to change was seen as a significant obstacle to the QI, as many midwives prefer to remain in their comfort zone and view changes as criticism.

When I did my Master’s. I found out a lot had changed … .but the people in the labour ward were not changing. They were the same midwives. They had not gone for further studies and they had not done any search on evidence-based practice. They were still doing the same things, and you hear them telling you – But this is what we have always done. (Informant #4)

However, the vast majority of the hospital staff participants described a will to adopt new solutions and evidence-based care and a wish for personal and organisational improvements. One doctor (informant #2) said, *‘We always have to check ourselves and find ways of improving the service.’*

Regulatory and academic representatives highlighted a potential barrier in staff hierarchies preventing midwives from practising within their scope. According to hospital staff, teamwork between midwives and doctors was, however, generally described as well functioning, and peer learning between junior and senior midwives was described as a facilitator for the upcoming midwife-led QI. Midwife #7 said, *‘We need to work together and learn from each other to provide the best care possible.’*

Midwives and doctors acknowledged the problem of having no shared goal of quality of midwifery standards of care. Also, bridging the gap between knowledge and action and lacking skills due to few opportunities for continued professional development were seen as barriers for the QI. To bridge this gap, participants suggested that onsite clinical education is essential. A facilitator for the upcoming QI intervention and an opportunity for knowledge sharing and updates on new guidelines is the weekly department meetings, a forum for midwives and doctors to report on the quality of care. Additional facilitators for a midwife-led QI at the hospital include midwives being engaged in education and outreach efforts, having prior experience in QI from other healthcare domains and having access to up-to-date information from professional midwifery associations. To further facilitate the midwife-led QI intervention, patience, structured approaches, and sufficient time were also seen as necessary to change current working methods.

All sectors described a compatibility between a midwife-led QI and their policies or plans. At the hospital, the intervention was described as a priority, which aligned with the hospital’s policies and quality assurance plans. The policy-level representative meant that midwives taking charge of QI efforts could be a critical means of bridging the gap between policy and practice and could increase the utilisation of hospital-based services. The policy-level representative further noted that a QI intervention on midwifery practises aligned with Agenda 2030 and was consistent with the human rights approach outlined in Uganda’s constitution. Respondents from academic institutions similarly mentioned that many midwifery practices, such as intrapartum support and women’s decision-making, were described in the national guidelines, even so they are still not often practised.

**Figure 1.**
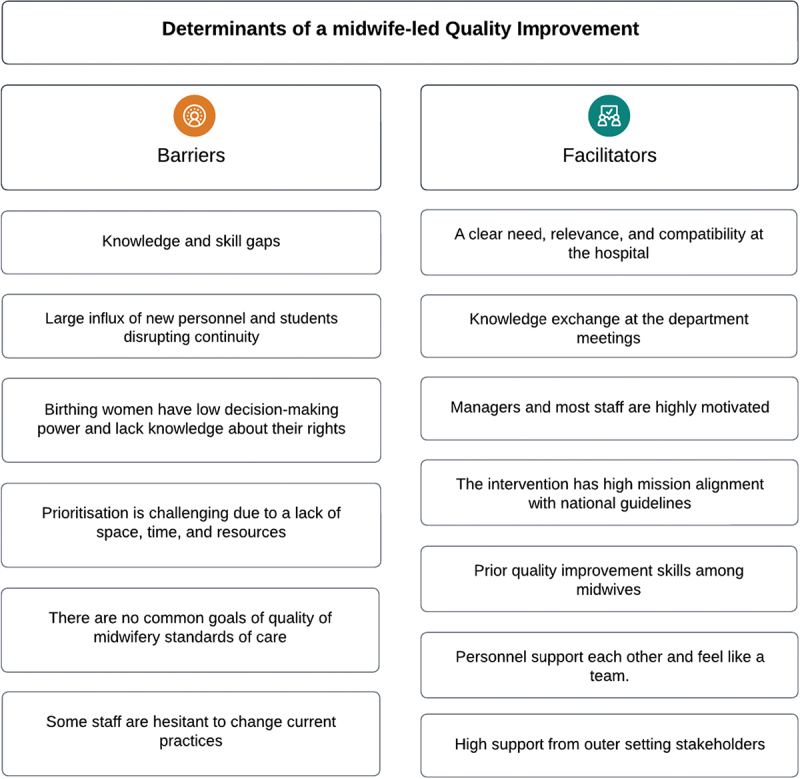
Determinants of a midwife-led quality improvement.

The academic institutions desired more influence and effective partnerships with clinical sites to promote evidence-based midwifery practices. One representative suggested that a more practical, hands-on approach to midwifery training would help increase the adoption of evidence-based midwifery practises. *‘The teachers mention different birth positions and support measures but cannot show how to practically use them’ (Informant #3)*. The academic institution representatives acknowledged their responsibility to make this change and envisioned collaborating with the future midwife-led QI team at the hospital to create a more cohesive training experience for students. The representative from the Professional Midwife Association emphasised that the midwife association could play a critical partner role in a midwife-led QI by empowering its members to push for changes in current practices. Further, the association meant that sustainable change must come from various sectors; educational institutions must provide practical skills training and advocate for the importance of quality midwifery practices, while policymakers should involve midwives as leaders in creating and implementing new guidelines. For a summary of determinants, see Figure 1.

### Third stage - co-creation workshops

New mothers brought up touchpoints for improving the quality of care related to their lack of decision-making power in the birthing process, including choosing their preferred birth position. However, the most common touchpoint among new mothers and their birth companions was to focus on improved intrapartum support, especially on respectful care, emotional support, encouragement, and information on procedures.

Midwives described touchpoints for midwifery practises such as improving skin-to-skin care, breastfeeding practises, triaging and information on postpartum danger signs before discharge. However, the most common touchpoint among midwives was improving women’s decision-making regarding using different birth positions to facilitate normal birth. During the midwives’ co-creation workshops, concerns were also raised regarding perineal support. The potential for adverse outcomes due to insufficient midwife skills in perineal protection while using various birth positions, coupled with the hospital’s relatively high rates of episiotomies and perineal tears, led to identifying the QI component – enhancing perineal protection.

In conclusion, three evidence-based midwifery practice areas were identified as priorities for the upcoming QI intervention.

1. Facilitating birth using dynamic birth positions

**Includes**: Giving birth on a birth chair, standing on all four, laying on the side, kneeling and knee-chest position

**Evidence**: Increases space in the pelvis, reduces medical interventions, shortens the first stage of labour, and enhances women’s comfort [32,33].

2. Perineal protection

**Includes**: Communicating with the woman, listening to the woman’s urge to push, slow 2–stepped birth of the baby’s head, perineal support with two hands, and avoiding routine episiotomies.

**Evidence**: Reduces tears, decreases pain, alleviates workload [32,33]

3. Enhanced quality of intrapartum support

**Includes**: Emotional support (caring attitude, positive and calming communication), physical support and comfort measures (touch, massage, hygiene), information and advice (listening to women’s views, giving instruction on breathing and relaxation and information about routines, procedures and progress) and advocacy (assisting in informed decision-making)

**Evidence**: Higher frequency of spontaneous vaginal deliveries, somewhat shorter birth, positive birth experience, fewer newborns with low five-minute Apgar score [32,33].

### Fourth stage - finalising the QI intervention design

#### Tailoring strategies, dose, temporality and roles

Based on the CFIR recommendations and guided by ERIC [26], strategies were selected to address the findings from the barriers and contextualised using the facilitators found in the second stage (see Table 2). Only initial strategies were included as the subsequent decisions were to be determined in the upcoming QI intervention.

**Table 2.** Initial intervention strategies based on the expert recommendations for implementing change’s list of recommended strategies [27].

Barriers targeted	Strategies	Contextualised recommendations using the identified facilitators	Actors
Knowledge and skills gaps among staff concerning evidence midwifery practises and national guidelines	Use train-the-trainer strategies Conduct educational meetings Develop and distribute educational material Promote network weaving	Train and educate designated clinicians to train others in the intervention. Make use of prior quality improvement skills and educational skills among midwives. Use practical, hands-on approaches to midwifery care. Develop, print and distribute educational material for staff and hold training sessions using the material. Build on existing relationships and networks with educational institutions, the regulation/policy sector, and the professional association to promote information-sharing, collaborative problem-solving, and a shared goal related to the intervention.	Midwize Ambassadours, support team, academic, policy and association representatives
Large influx of new personnel and students disrupts the continuity	Develop and distribute educational materials	Develop and distribute educational material and include QI components training in the existing learning structures.	Midwize Ambassadours, support team
Birthing women have low decision-making power and lack knowledge about their rights to supportive care	Develop and distribute educational material	Determine the information for pregnant women and companions at antenatal care and hospital admission, ensuring effective and inclusive distribution.	Midwize Ambassadours, support team
Prioritising is challenging due to a lack of space, time, and resources	Mandate change Use existing structures Revise professional roles	Have leadership declare the need, relevance and compatibility of the intervention and their determination to implement it. During training and discussions, emphasise that evidence-based midwifery practises can be done within existing structures without much additional material resources. Involve students and birth companions in providing supportive care.	Hospital managers, Midwize Ambassadours, policy sector representative
No common goals of quality of midwifery standards of care	Conduct local consensus discussions Use existing structures	Include midwives from different hospital departments (ANC/Admission/Labour) and other relevant staff in discussions and goal-setting meetings. Use weekly department meetings to share, discuss and remind the identified common goals of quality of midwifery standards.	Midwize Ambassadours
Some staff are hesitant to change current practices.	Identify early adopters Use train-the-trainer strategies Mandate change Use existing structures	Involve the managers and staff that are highly motivated. Train and educate designated clinicians to train others in the intervention. Use practical, hands-on approaches to midwifery care. Have leadership declare and explain the intervention’s alignment with national guidelines, need, relevance and compatibility, and their determination to implement it. Knowledge exchange at weekly department meetings and WhatsApp groups. Make use of the existing team spirit.	Midwize Ambassadours, hospital Managers, policy sector representative

Midwize Ambassadors were selected by the research team and a midwife manager based on their willingness to participate, current position, leadership skills, and ability to influence colleagues. To cover all shifts and the relevant departments at the hospital, a total of seven Ambassadors were considered necessary. Their roles in the QI were initially discussed and decided based on the intervention strategies in Table 2. The QI would start with more guidance from the support team consisting of researchers with midwife-led QI competence (JB, HL) and a clinical midwifery teacher and gradually transition to being entirely led by the Midwize Ambassador group.

The Midwize Ambassadors and support team jointly discussed and agreed on the temporality and dose of the intervention based on the need and the feasibility in the specific setting. A three-day onsite train-the-trainers programme, led by the support team, was to be held, and each Ambassador would receive eight hours of individual clinical training and support at the labour ward. Train-the-trainer support would be provided, including weekly 60–minute online sessions and bi-monthly physical meetings with the support team. Video recordings, suggested readings and QI templates would be accessible online for the Ambassadors.

## Discussion

Our study, which seeks to collaboratively design a QI intervention to bridge the gap between evidence and practice in midwifery, outlines four key stages. In the first stage, the core elements of the QI intervention are defined, including the leadership of ‘Midwize Ambassadors’ and a focus on evidence-based practices using a ‘train the trainers’ approach. In the second stage, we evaluated needs and context, underscoring the importance of empowering women in childbirth decisions, fostering shared goals within the team, promoting current knowledge exchange at the department, and boosting staff motivation. In the third stage, co-creation workshops identified key priorities for the QI. These included facilitating dynamic birth positions, enhancing perineal protection, and improving the quality of intrapartum support based on evidence-based practices. In the fourth stage, the finalisation of the QI intervention design involved tailoring strategies and assigning roles to Midwize Ambassadors with the aim of transitioning full leadership to this group over time. Learning from these four stages, this QI co-creation approach can help address healthcare delivery challenges in different settings.

Most healthcare interventions only focus on changing service providers’ behaviours despite their limited impact and challenges in sustainability and scalability [34]. In this study, co-creating with multiple sectors, rather than only service providers, helped highlight important areas and structures to be considered in the upcoming intervention. For example, new mothers and birth companions identified enhanced intrapartum support as a specific component. This finding exemplifies how end-user engagement, perspectives and experiences can shape an intervention and uncover critical aspects that might have been overlooked otherwise. Previous studies additionally show that involving end-users fosters trust and collaboration and promotes empowerment, awareness, and a sense of social contribution [32]. These factors can be seen as impacting care-seeking behaviours and health outcomes, further underscoring the importance of involving end-users in the process [35,36]. In line with previous literature, our interpretation of this finding is that future co-created interventions may benefit from multi-sectoral engagement, especially highlighting end-user engagement and that such factors must be considered to ensure the long-term sustainability of an intervention [34,37].

In the study, the midwives at the clinical site demonstrated an unutilised desire and enthusiasm for shaping and leading their agenda while being supported by managers, doctors, policymakers and academia. Overall, previous studies show that including midwives as leaders across different levels, from high-level decision-makers to quality improvement leaders, has profound effects: motivating individuals, improving care quality, empowering the healthcare workforce and allocating funds to women’s and children’s health [38–40]. Consequently, our findings and previous research suggest that allowing midwives to take up leadership roles can pave the way for creating relevant interventions to address healthcare challenges. Nonetheless, a persistent inequality remains in leadership opportunities for midwives and women at all healthcare system levels [40]. This imbalance underscores the necessity of the upcoming midwife-led QI initiative and its co-creation process by providing an opportunity to tap into midwives’ expertise and first-hand experiences to drive advancements. By embracing midwives’ insights and empowering them to shape the future, we can push transformative improvements of the healthcare system forwards.

## Strengths and limitations

One notable strength of this study is the use of CFIR to guide the creation of the intervention. This facilitated a structured analysis of needs, context and determinants and guidance on all four stages of the process. Adopting the CFIR framework provided advantages, given its comprehensive nature. However, it also presented a limitation as it was not feasible to address all 39 constructs during data collection, potentially leaving out valuable insights for the analysis [20]. To mitigate this limitation, the researcher group engaged in iterative discussions to identify the most relevant construct for the specific context.

The authors of this study represent a spectrum of disciplines associated with maternal and newborn health, including midwifery, quality improvement expertise, and obstetrics, stemming from different countries. This diversity enriches the study by offering a multifaceted, cross-national, and multi-expertise perspective. However, unconscious bias in interpreting the empirical data might have been introduced by the authors who are not from the study context. To reduce this potential bias, the Categorisation matrix based on CFIR laid out by the authors was adhered to according to the analysing method [29]. We then followed an iterative process where the analysis was reviewed and discussed with all authors who possessed situated cultural knowledge and extensive research experience from the study country.

## Conclusion

This study provides an account of the co-creation process employed to develop a midwife-led QI intervention using the CFIR framework. By shedding light on how to include stakeholders from relevant sectors and having midwives and end-users at the core of the co-creation process, this study serves as a practical resource for advancing the field of midwifery and promoting patient-centred healthcare improvements. The findings can be used as an inspiration for future quality improvement efforts in other maternity care settings.

## Paper context

This study provides a practical example of a co-creation process for developing a quality improvement intervention on evidence-based midwifery practises. The intervention becomes relevant and compatible with guidelines and needs through the co-creation approach used in this study, bridging the evidence-to-action gap in maternal healthcare. It is recommended that more maternal healthcare interventions be co-created and that the finding in this study is used as an inspiration in future midwife-led quality improvement efforts.
